# Transcriptomic Profiling of mRNA and lncRNA During the Developmental Transition from Spores to Mycelia in *Penicillium digitatum*

**DOI:** 10.3390/microorganisms13122879

**Published:** 2025-12-18

**Authors:** Ting Zhou, Yajie Yang, Fei Wang, Linqian Liang, Ziqi Zhang, Heru Dong, Zhaocheng Jiang, Pengcheng Zhang, Tongfei Lai

**Affiliations:** College of Life and Environmental Science, Hangzhou Normal University, Hangzhou 310036, China; 20100061@hznu.edu.cn (T.Z.); 2024111010034@stu.hznu.edu.cn (Y.Y.); 2025111010051@stu.hznu.edu.cn (F.W.); 2025112010055@stu.hznu.edu.cn (L.L.); 2023210301069@stu.hznu.edu.cn (Z.Z.); 2023210301042@stu.hznu.edu.cn (H.D.); 2023210301051@stu.hznu.edu.cn (Z.J.)

**Keywords:** green mold, growth, transcriptome, lncRNAs

## Abstract

The fungus *Penicillium digitatum* causes citrus green mold, a major postharvest disease. Understanding the molecular mechanisms underlying its development is crucial for devising effective control strategies. In this study, we performed a comprehensive transcriptomic analysis of *P. digitatum* across three key developmental stages: spores, germinated spores, and mycelia. A total of 2175 novel mRNAs, 3957 novel long non-coding RNAs (lncRNAs), and 144 circular RNAs (circRNAs) were identified in *P. digitatum*. Genetic variation analysis revealed 12,396 Insertion-Ddeletion and 23,264 single nucleotide polymorphisms, with their prevalence decreasing as development progressed. The expression levels, temporal expression patterns and significant differences in mRNAs and lncRNAs across different developmental stages were also observed. Functional enrichment analysis of differentially expressed mRNAs and differentially expressed lncRNA target genes highlighted key biological processes and pathways associated with macromolecular metabolism, signal transduction, DNA replication, and reactive oxygen species scavenging. Additionally, differential expression analysis explored the potential interactions between differentially expressed lncRNAs and their target genes, as well as those between lncRNAs and circRNAs. Our findings provide valuable insights into the complex regulatory networks underpinning the development and pathogenicity of *P. digitatum*, offering a foundation for future research aimed at controlling green mold.

## 1. Introduction

*Penicillium digitatum*, a prominent member of the *Penicillium* genus, is the primary causative agent of green mold, a devastating postharvest disease of citrus fruits responsible for substantial economic losses globally [1]. Beyond its impact on fruit quality, *P. digitatum* produces mycotoxins, such as tryptoquialanines, which pose significant health risks to consumers [2]. Infection typically occurs through surface wounds or bruises on citrus fruit, where the fungus readily establishes itself. Following invasion, *P. digitatum* produces a characteristic greenish spore mass, facilitating rapid dissemination to adjacent fruits, particularly during storage or transit. This aggressive spread underscores its role as a major contributor to postharvest losses in the citrus industry [3]. Current management strategies rely on a multi-pronged approach, including chemical fungicides, optimized handling and storage protocols, and the development of resistant citrus varieties [4]. However, overreliance on fungicides has triggered the emergence of resistant *P. digitatum* strains and sparked concerns regarding environmental and human health [5]. Consequently, the development of innovative, sustainable control methods is critical for the long-term viability of the citrus industry.

A comprehensive understanding of the molecular mechanisms governing *P. digitatum*’s life cycle is essential for devising effective control strategies. To date, numerous key genes associated with fungal development and pathogenicity have been identified. For instance, chitin synthase genes (*PdigCHSI*-*VII*) [6,7], *protein O-mannosyltransferase 2* (*Pdpmt2*) [8], glucosylceramides synthase genes (*PdGcs1, PdMit1*) [9,10], APSES transcription factor gene (*PdStuA*) [11], glycinamide ribonucleotide-transferase gene (*Pdgart*) [12], cell polarity protein gene (*PdMesA*) [13], and H^+^-ATPase genes [14] are essential for infection structure formation and spore development of *P. digitatum*. Polygalacturonases genes (*Pdpg1*, *Pdpg2*, *Pdpgax*, and *Pdpnl1*) [15,16] and xyloglucan-specific endo-β-1,4-glucanase gene (*PdXEG1*) [17] mediate cell wall breakdown, releasing carbon sources essential for fungal growth post-invasion. Signaling components such as *PdpacC* (pH signaling) [18], *PdMpkB* and *Pdslt2* (MAPK pathways) [19,20], *Pdac1* and *PdGpaA* (cAMP signaling) [21,22], *PdCrz1* (Ca^2+^/calmodulin pathway) [23], and *PdMFS2-5* (major facilitator superfamily transporters) [24] are required for full pathogenicity. Despite these advances, the molecular regulatory networks underlying *P. digitatum*’s development and virulence remain incompletely understood.

The life cycle of *P. digitatum* is exclusively asexual and begins with airborne spores dispersing and landing on hosts. Under optimal conditions, spores germinate, form germ tubes, and develop into invasive hyphae. Subsequently, the mycelia differentiate into conidiophores that produce new conidia. In this study, we focused on three critical developmental stages: spores (Pd-S), germinated spores (Pd-G), and mycelia (Pd-M). The spores are the cornerstone of pathogenicity—ensuring resilience, widespread dissemination, efficient host invasion, and relentless disease cycles. The germinated spores are biologically indispensable as the bridge between spores and mycelia. They execute host recognition, penetration, and early virulence. The mycelia are responsible for destructive colonization and reproduction. They secrete macerating enzymes and organic acids, degrading host cell walls and tissues, causing soft rot decay and facilitating nutrient absorption. Crucially, the mycelium also develops conidiophores, producing massive quantities of new spores for secondary spread and perpetuating the disease cycle.

Transcriptome sequencing has revolutionized the study of fungal biology by providing a comprehensive, high-resolution snapshot of gene expression at a given moment. Beyond profiling protein-coding mRNAs, transcriptome analysis is instrumental in characterizing a suite of non-coding RNAs and genetic variations with profound regulatory roles. Long non-coding RNAs (lncRNAs), defined as transcripts longer than 200 nucleotides without protein-coding potential, can act as epigenetic regulators, molecular scaffolds, or competitive endogenous RNAs to fine-tune the expression of pathogenicity genes [25,26]. Circular RNAs (circRNAs) are a unique class of covalently closed-loop RNAs that are highly stable and can function as microRNA sponges, thereby sequestering these regulatory molecules and influencing mRNA stability and translation [27,28]. MicroRNAs (miRNAs) are short non-coding RNAs that typically bind to target mRNAs to induce their degradation or translational repression, representing a crucial layer of post-transcriptional control [29,30]. At the DNA level, the analysis of Insertions and Deletions (Indels), which are small additions or losses of nucleotides, and Single Nucleotide Polymorphisms (SNPs), which are variations at a single base pair, can identify genetic mutations that confer fungicide resistance or alter virulence. Furthermore, Alternative Splicing (AS) is a mechanism whereby a single gene can produce multiple mRNA isoforms, greatly expanding the functional proteome and allowing the fungus to rapidly generate different protein variants suited for specific developmental stages or environmental challenges.

Therefore, leveraging the well-annotated *P. digitatum* genome and advances in RNA sequencing [31,32], we employed high-throughput transcriptomics to capture the dynamic and coordinated gene expression programs governing critical developmental transitions—from spores to germinated spores and ultimately to invasive mycelia. This approach allowed us to identify key virulence factors, developmental regulators, and novel regulatory networks, thereby moving beyond the static genome to reveal the functional genome in action. The integrated study of these elements provides a holistic view of the complex regulatory networks driving fungal growth and virulence, thus unveiling potential targets for novel, RNA-level control strategies to mitigate postharvest losses.

## 2. Materials and Methods

### 2.1. Pathogen and Morphological Observation

*Penicillium digitatum* was obtained from a naturally infected orange (*Citrus sinensis*) exhibiting characteristic green mold symptoms. A segment of necrotic tissue (approximately 0.3 × 0.3 cm^2^) containing fungal reproductive structures was aseptically isolated from an infected fruit. It was surface-sterilized with 75% ethanol for 2 min, rinsed thoroughly with sterile distilled water, and air-dried. Subsequently, it was transferred onto potato dextrose agar (PDA; Solarbio, Beijing, China) medium for culture. After 7 days of culturing, hyphal tips were transferred to PDA and cultured at 25 °C under 16 h photoperiods. To confirm the genetic identity of the fungal isolate, total genomic DNA was extracted from a monoconidial culture using the DNeasy Plant Mini Kit (Qiagen, Hilden, Germany). Subsequently, the internal transcribed spacer (ITS) and beta-tubulin (β-tubulin) gene regions were amplified by polymerase chain reaction (PCR) with the primer pairs ITS1/ITS4, ITS4/ITS5, and BT2a/BT2b, respectively, according to previously described methods (Table S1) [33,34]. Following amplification, the PCR products were purified and sequenced by Sangon Biotech Co., Ltd. (Shanghai, China). A BLAST search was conducted against the non-redundant nucleotide database using the NCBI online platform (http://blast.ncbi.nlm.nih.gov/Blast.cgi (accessed on 10 July 2024)). A circular phylogenetic tree was constructed from the pairwise alignments of the top 20 BLAST hits using the Fast Minimum Evolution (FastME) method [35]. The score was a numerical value that quantifies the quality of local alignment between the query sequence and a database sequence. The maximum allowed fraction of mismatched bases in the aligned region between any pair of sequences (Max Seq Difference) was set to 0.75. The distance between sequences used for tree generation estimates the expected fraction of base substitutions per site, derived from the observed fraction of mismatched bases in the aligned region.

**Figure 1:**
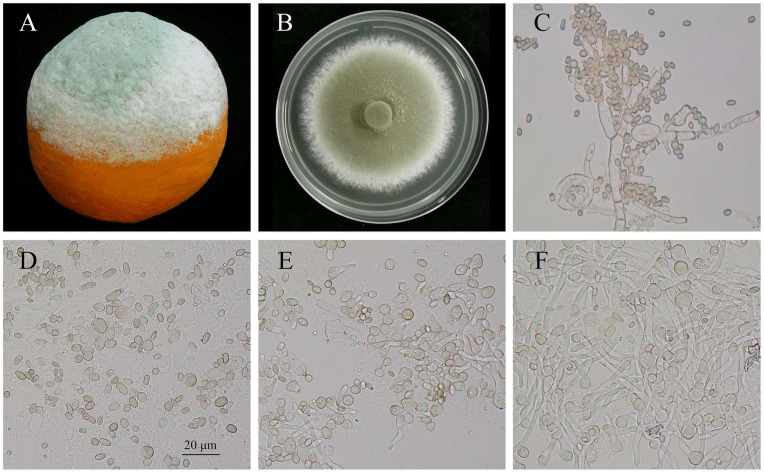
The phenotype of *Penicillium digitatum*. (**A**) Green mold of *Citrus sinensis* (L.) Osbeck; (**B**) Colonial morphology of *P. digitatum*; (**C**) Spores and mycelia of *P. digitatum*; (**D**–**F**) The phenotypic changes in *P. digitatum* spores after 4 h (**D**), 8 h (**E**), and 12 h (**F**) of culturing in PDB.

An aliquot of a fresh spore suspension was introduced into 100 mL of potato dextrose broth (PDB; Solarbio, Beijing, China) medium, adjusting the concentration to 1.0 × 10^6^ spores/L. Incubation was carried out at 25 °C with agitation at 200 rpm. Samples representing different developmental stages, spores (Pd-S, 4 h), germinated spores (Pd-G, 8 h), and mycelia (Pd-M, 12 h) (Figure 1D–F), were harvested via centrifugation and subjected to two washes with sterile distilled water. These samples were then flash-frozen in liquid nitrogen and maintained at −80 °C for future use. For documentation, colony morphology and diseased fruit exhibiting blue mold symptoms were photographed with a Canon EOS 60D camera (Canon, Tokyo, Japan) (Figure 1A,B). Fungal structures were examined and imaged using a Nikon Eclipse Ni-U microscope (Nikon, Tokyo, Japan) (Figure 1C–F).

### 2.2. Detection of Fungal Physiological Characteristics

To monitor germination, *P. digitatum* spores were cultured in PDB (1.0 × 10^6^ spores/L) at 25 °C with shaking (200 rpm), and germination percentage along with germ tube length were measured microscopically every two hours from 4 to 12 h. For biomass analysis, mycelia from 100 mL cultures were collected after 24, 48, and 72 h. The fresh weight was measured post-centrifugation, and the dry weight was obtained after drying the mycelia to constant weight at 60 °C.

The colony and lesion expansion of *P. digitatum* on PDA plates and citrus fruits was determined daily. Sporulation of *P. digitatum* was assessed by spreading 100 µL of a spore suspension (about 100 spores) onto potato dextrose agar (PDA) in 9 cm Petri dishes. Following static incubation at 25 °C, spores were harvested at 3, 6, 9, and 12 days using a 0.1% Tween-80 solution and quantified with a hemocytometer.

### 2.3. Sample Preparation and qRT-PCR Validation

The selection of activated spores over dormant spores for transcriptomic analysis is crucial because dormant spores are metabolically quiescent, with a transcriptome geared for survival rather than pathogenesis. The *P. digitatum* samples at different developmental stages with a biological repeat (Pd-S-1, Pd-S-2, Pd-G-1, Pd-G-2, Pd-M-1, Pd-M-2) were prepared as described above. Each sample was pooled from three independently cultured specimens and was divided into two parts. One part was used for transcriptomic analysis, and the other was used for quantitative real-time PCR (qRT-PCR) validation. Total RNA was extracted with TRIzol Reagent (Invitrogen, Carlsbad, CA, USA), and cDNA was synthesized from the RNA using a FastQuant RT Kit (Tiangen Biotech, Beijing, China) according to the manufacturer’s instructions. Quantitative real-time PCR was then carried out on a CFX96 system (Bio-Rad, Hercules, CA, USA) using the 2× Ultro SYBR mixture (CW, Beijing, China) to determine relative gene expression levels. The information on primer pairs for specific genes was provided in Table S2. The expression level of *β-tubulin* was used for normalization, and the expression change in each gene was determined using the 2 (^−ΔΔCt^) analysis method.

### 2.4. Transcriptomic Analysis

The technological service of transcriptome analysis was provided by Beijing Genomics Institute (BGI) Co., Ltd. (Beijing, China). The RNA extraction process was described in technical specification BGI-NBS-TQ-RNA-002 (Ver. A0). Total RNAs were qualified and quantified using an Agilent 2100 Bioanalyzer (Agilent, Santa Clara, CA, USA). The lncRNA library preparation was described in the technical specification (https://www.yuque.com/yangyulan-ayaeq/oupzan/aa2d55 (accessed on 10 December 2024). The main steps included sample quality control, rRNA removal, RNA fragmentation, cDNA synthesis, end repair, add A and adaptor ligation, PCR amplification, circularization of PCR products, and DNA nanoball synthesis. Subsequently, all libraries were sequenced on an Illumina Hiseq 2500 platform (Illumina, San Diego, CA, USA) following the standard experimental operation protocol.

### 2.5. Bioinformatic Analysis

To eliminate the impact of sample quality and species, low-quality reads, duplication reads, adapters, and reads with high content of N bases were first removed from raw reads using SOAPnuke (v1.5.2). The clean reads could be acquired in the Sequence Read Archive (SRA) database with the accession ID PRJNA1302482. Following alignment of the reads to the *P. digitatum* reference genome (GenBank: GCA_016767815.1) using HISAT2 (v2.0.4) [36], transcripts were reconstructed with StringTie (v1.0.4) [37]. These assemblies were then compared to known mRNA and lncRNA annotations using Cufflinks (v2.2.1). The coding potential of novel transcripts was assessed with a comprehensive approach utilizing CPC (v0.9-r2) [38], txcdsPredict (v1.0), CNCI (v1.0) [39], and the Pfam database [40]. For gene expression quantification, clean reads were mapped back to the reference gene set using Bowtie2 (v2.2.5) [41], and expression levels were calculated with RSEM (v1.2.12) [42]. Fragments per kilobase of the exon model per million mapped fragments (FPKM) was used to standardize gene expression levels. The software DEGseq (v1.60.0) was used for intergroup difference analysis [43]. A statistical model based on the negative binomial distribution and likelihood ratio test was employed to account for biological variability in read counts between groups at different developmental stages. To correct for multiple hypothesis testing, DEGseq applies a false discovery rate (FDR) adjustment. The filtration conditions of differentially expressed genes (DEGs) were as follows: fold change ≥ 2.0 and FDR ≤ 0.001. The software pheatmap (v1.0.8) was used to cluster genes based on expression levels, and the Mfuzz (v3.21) was used for gene-expression time-course clustering [44].

Functional annotation of the assembled mRNAs was conducted through BLAST (v2.2.23) searches against several databases, including Non-Redundant Protein Sequence Database (NR), Nucleotide Sequence Database (NT), Clusters of Orthologous Genes Database (COG), Swiss-Prot database (SwissProt), and Kyoto Encyclopedia of Genes and Genomes Database (KEGG). GO annotations were assigned by integrating results from Blast2GO (v2.5.0) and the NR database, while protein domains and families were identified using InterProScan (v5.11-51.0) [45,46]. Subsequently, enrichment analysis for these genes was performed based on the GO and KEGG classifications. The significance of enrichment for each functional category was assessed by its *p*-value, with a smaller *p*-value indicating a greater abundance of candidate genes in that module. Terms with a False Discovery Rate (FDR)-adjusted *p*-value ≤ 0.01 were considered significantly enriched.

The GATK (3.4-0) was used to analyze the Single-Nucleotide Polymorphisms (SNPs) and Insertion-Deletion (INDEL) for each sample [47]. The ASprofile (v1.0.4) was used to classify alternative splicing (AS) events of transcripts [48]. The pipeline began by aligning RNA-seq reads to the reference genome, followed by generating transcripts as described above. ASprofile then compared these transcript structures to the reference annotation to identify and categorize AS events into common types and calculated splicing ratios for each event, quantifying the relative abundance of different isoforms. The rMATS (v4.3.0) was used to detect differentially spliced genes (DSGs) corresponding to all major types of patterns [49]. The CIRI (v2.1.1) was used to identify the circular RNAs (circRNAs) and estimate the relationships between the circRNAs and lncRNAs depending on the location relationship [50].

The mRNAs adjacent to the lncRNAs were selected as target genes if the Spearman and Pearson correlation coefficient of lncRNA and mRNA was ≥0.6. The lncRNA in the 10 k upstream or 20 k downstream of the mRNA was determined as *cis*-acting. Beyond this range, the RNAplex (v0.2) was used for analysis of the binding energy of the lncRNA and mRNA [51]. It was determined as *trans*-acting if the binding energy was ≤30. The overlap of the lncRNAs and their target genes was classified. The NFERNAL (v1.1) aligned the lncRNAs to the Rfam database to annotate the family of lncRNAs [52]. In addition, the relationships between differentially expressed (DE) lncRNAs and DE-lncRNA target genes were determined by differential expression analysis, and the networks were displayed by interaction diagrams.

### 2.6. Statistical Analysis

Data were subjected to statistical analysis in Microsoft Excel 2013 (v15.0.5589.1000). One-way analysis of variance (ANOVA) was employed to assess differences between multiple means. The threshold for statistical significance was established at *p* < 0.05.

## 3. Results

### 3.1. Morphological Characteristics of P. digitatum Across Developmental Stages

The *P. digitatum* used in this study can lead to typical green mold symptoms in citrus fruit. At the initial infection stage, the discolored, water-soaked spots appear, and the lesion expands rapidly, forming a circular and sunken decayed area. Subsequently, the white, fluffy mycelia spread over the fruit surface, and olive-green to grayish-green powdery spores in concentric rings were produced, finally forming a thick and dusty layer (Figure 1). On the PDA plate, white mycelia spread radially, forming a circular and fluffy colony in the early stage. Then, the colony covered with mass spores showed slight wrinkling, and the outer zone remained with white active hyphae within 9 d. The reverse side was yellow-brown to orange-brown. The phialides with flask-shaped produce branched and smooth-walled conidia (spores). Amplification with universal primer pairs yielded three distinct fragments: 604 bp (with ITS1/ITS4), 5894 bp (with ITS4/ITS5), and 1393 bp (with BT2a/BT2b) (Figure S1, Table S1). The sequences of the fragments amplified by the two primer pairs, ITS1/ITS4 and ITS4/ITS5, have been deposited in the public database GenBank with the accession numbers PX658547 and PX658549, respectively. Phylogenetic analysis revealed that the isolate was clustered within *Penicillium digitatum*, complementing the morphological identification (Figure S2). MegaBLAST analysis revealed that the ITS sequences shared the highest nucleotide identity (99.49% and 100%, respectively) with *Penicillium digitatum* strain 74. Similarly, the Beta-tubulin (BT2a/BT2b) sequence showed 99.86% identity to *Penicillium digitatum* strain Pd27.

In PDB medium, the spherical or subglobose spores absorbed nutrients and enlarged in size within 4 h. Although activated from dormancy, the differentiation and polar growth of most spores were not detected. By 8 h, the germination rate of spores was over 70% and the germ tube was clearly visible. After 12 h of culturing, the spores had developed into typical filamentous mycelia. During 24 h to 72 h, the hyphae branched, extended, and accumulated rapidly (Figure 1 and Figure S3). The spores’ germination and filamentous mycelia formation in *P. digitatum* were critical for its lifecycle, pathogenicity, and ecological impact.

### 3.2. Transcriptome Assembly Annotation

**Figure 2:**
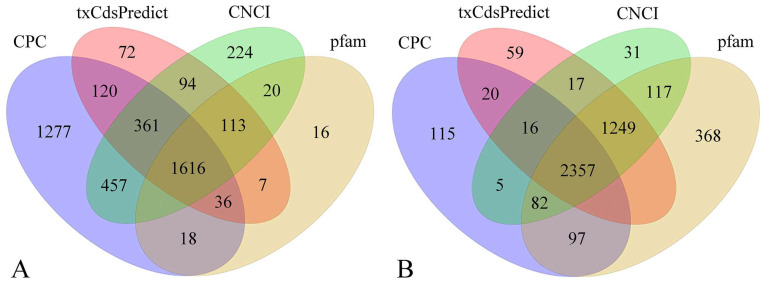
Venn diagrams showing predicted mRNAs (**A**) and lncRNAs (**B**) identified across the developmental stages of *Penicillium digitatum*. The different colors represent different prediction methods.

To capture the dynamic and sequential gene expression programs driving infection, we profiled *P. digitatum* at 4 h, 8 h, and 12 h time points post-culturing. This series represents a critical developmental continuum: the 4 h spores are in the early activation and germination stage, initiating host recognition and penetration programs; the 8 h spores exhibit active germ tube elongation and the robust expression of early virulence factors; and the 12 h mycelia are engaged in destructive colonization and nutrient acquisition. Through the Illumina HiSeq system, each sample produces about 10.83 GB of data. A total of 13,884 transcripts were identified across the six samples, with an average genomic mapping rate of 67.83% (Table S3). After coding capacity prediction, 3957 novel lncRNAs, 2175 novel mRNA, and 7752 known mRNAs were distinguished (Figure 2, Tables S4–S6). The annotation of mRNA genes in all samples was performed in NR, GO, KEGG, and COG databases (Figure S4, Tables S7–S10). Additionally, the predicted lncRNAs were classified into different families depending on the common ancestor at the evolutionary level. The number of lncRNAs within RF01306, RF01675, RF00005, and RF00519 families was relatively large (Figure S5, Table S11).

### 3.3. SNP and InDel Analysis

**Figure 3:**
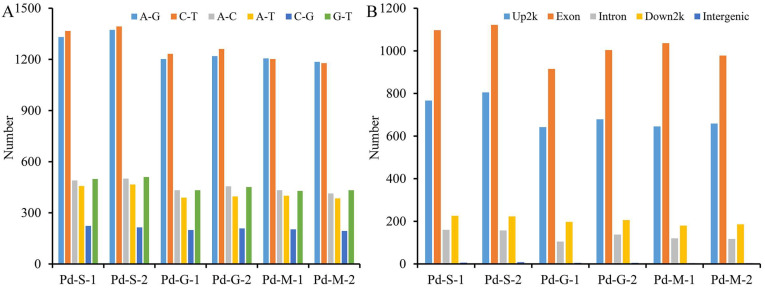
Single-nucleotide polymorphisms (**A**) and Insertion-Deletion statistics analysis (**B**) in *Penicillium digitatum* at the different developmental stages. A-G, C-T, A-T, C-G, and G-T indicate the SNP type of A to G, C to T, A to T, C to G, and G to T, respectively. Up2K Exon, Intron, Down2K and Intergenic indicate annotation to upstream 2k region of gene, exon region, intron region, downstream 2k region of gene, and gene intergenic region, respectively. Pd-S, Pd-G, or Pd-M represent spores germinated spores, or mycelia, respectively. Each number following represents an independent biological replicate.

SNP is a variation in a single nucleotide that occurs variation at DNA sequences, resulting in the diversity of genomes of individuals, including the transitions and transversions. In the present study, a higher frequency of transitions compared to transversions was observed in every sample, and the cytosine to guanine transversion type was the least abundant. Meanwhile, with extended culture time, the SNP count showed a progressive decline in *P. digtatum* (Figure 3A, Table S12).

An InDel is the addition or removal of a small number of bases in a DNA sequence. Similarly to the pattern of SNPs, ranked by quantity from highest to lowest, the distribution of the InDels was as follows: exons, upstream 2 kb of genes, downstream 2 kb of genes, introns, and intergenic regions in all samples. The number of InDels in the spores was also significantly higher than that in the germinated spores or mecylia (Figure 3B, Table S13).

### 3.4. AS and DSG Analysis

**Figure 4:**
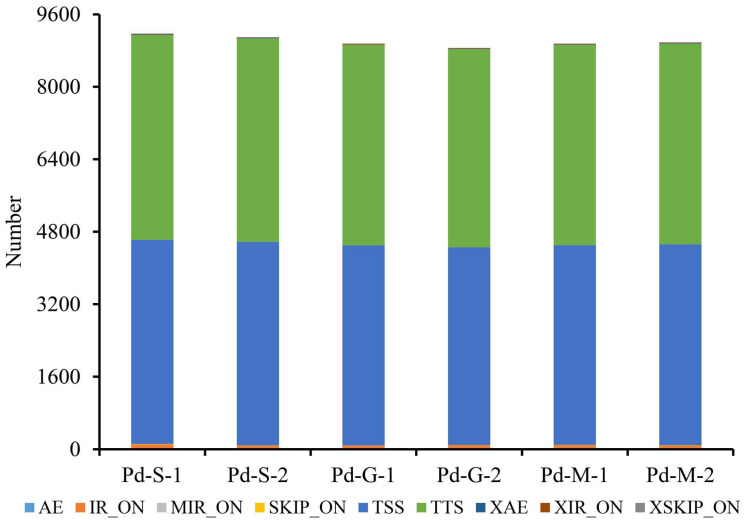
The alternative splicing statistics of mRNA in *Penicillium digitatum* at different developmental stages. AE: Alternative exon ends; IR: intron retention (IR_ON); MIR: multi-IR (MIR_ON); SKIP: skipped exon (SKIP_ON); TSS: alternative 5′ first exon (transcription start site); TTS: alternative 3′ last exon (transcription terminal site); XAE: approximate AE; XIR: approximate IR (XIR_ON); XSKIP: approximate SKIP (XSKIP_ON).

Alternative splicing is a regulated process in eukaryotic gene expression where a single pre-mRNA transcript can be spliced in multiple ways to produce different mature mRNA isoforms, often encoding distinct protein variants with diverse functions. More than 8800 ASs were detected in each sample, and no significant differences in abundance were observed across different sample groups. Among them, over 98.4% of identified ASs belonged to TSS (alternative 5′ first exon) and TTS (alternative 3′ last exon). About 0.7% of ASs were classified as IR_ON (intron retention) (Figure 4, Table S14).

A total of 317 alternative splicing events were identified between spores and germinated spores, consisting of 7 mutually exclusive exons (MXE) and 308 skipped exons (SE). Similarly, 279 events were detected between spores and mycelia, including 11 MXE and 268 SE. However, statistical tests showed non-significant *p*-values (FDR > 0.1). Nevertheless, the GO enrichment analysis of the possible differentially spliced genes (DSGs) was performed and provided in Figure S6.

### 3.5. Differential Expression Analysis and RT-qPCR Validation

**Figure 5:**
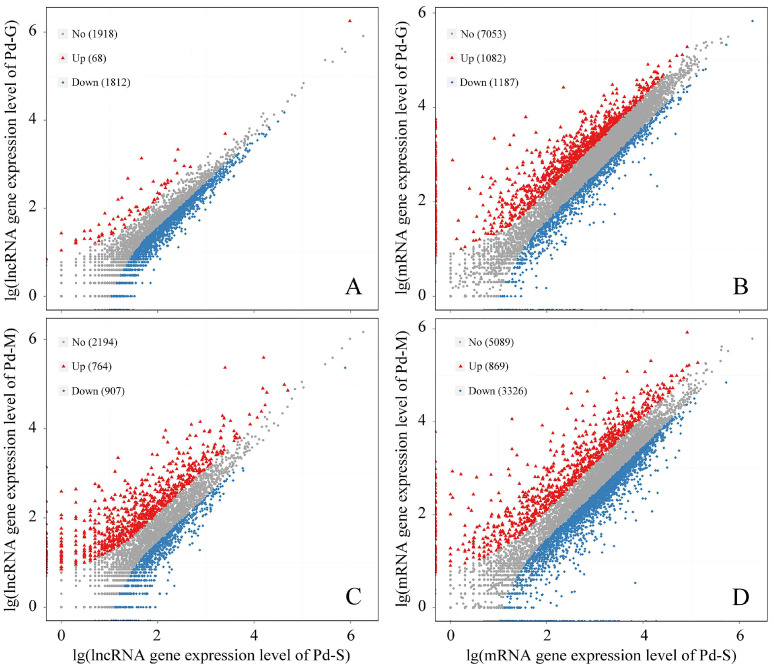
Scatter plot of stage-specific mRNA and lncRNA expression in *Penicillium digitatum*. (**A**) DE-lncRNAs between Pd-S and Pd-G; (**B**) DE-mRNAs between Pd-S and Pd-G; (**C**) DE-lncRNAs between Pd-S and Pd-M; (**D**) DE-lncRNAs between Pd-S and Pd-M. Red triangles, blue squares and gray dots indicate up-regulated, down-regulated and no differentially expressed genes.

**Figure 6:**
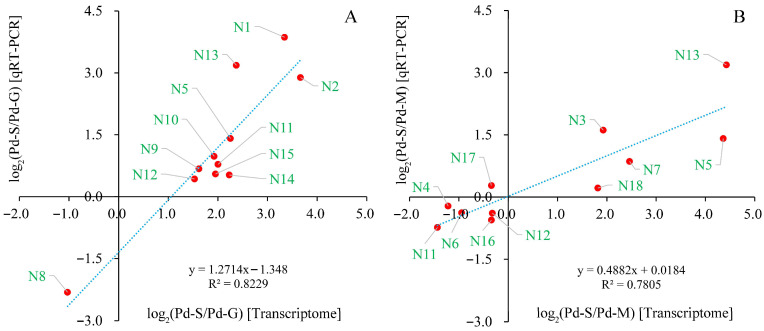
Regression analysis of expression levels exposed by transcriptomic sequencing and qRT-PCR via randomly selective DEGs. (**A**) DEGs between Pd-S and Pd-G; (**B**) DEGs between Pd-S and Pd-M. The *x* axis indicates log_2_ (fold change in DEGs) acquired from transcriptomic sequencing. The *y* axis indicates log_2_ (fold change in DEGs) acquired from qRT-PCR. Red dots depict individual differentially expressed genes (DEGs), and the blue dashed line represents the optimum imitative straight line, with the correlation coefficient indicated as R. Details for the DEGs labeled N1 to N18 are provided in Table S2.

The expression profiles of all predicted mRNAs and lncRNAs are summarized in Figure S7 and Table S15, with their temporal dynamics depicted in Figure S8. Substantial expression variation was observed across the developmental stages of *P. digitatum*. Specifically, a comparison between spores and germinated spores identified 2269 differentially expressed mRNAs (1082 up, 1187 down) and 1880 DE-lncRNAs (68 up, 1812 down). Likewise, the comparison between spores and mycelia revealed 4095 DE-mRNAs (869 up, 3326 down) and 1671 DE-lncRNAs (764 up, 907 down) (Figure 5, Table S16). The clustering and of DE-mRNAs and DE-lncRNAs were shown in Figure S9. Compared with expression levels of 18 randomly selected DEGs between transcriptomic sequencing and RT-qPCT analysis, the coefficients of determination (R^2^) in linear regression were 0.8229 and 0.7805 for two comparison groups (Figure 6). The higher correlation implied the higher reliability and biological relevance in the transcriptomic data.

### 3.6. Functional Enrichment Analysis

**Figure 7:**
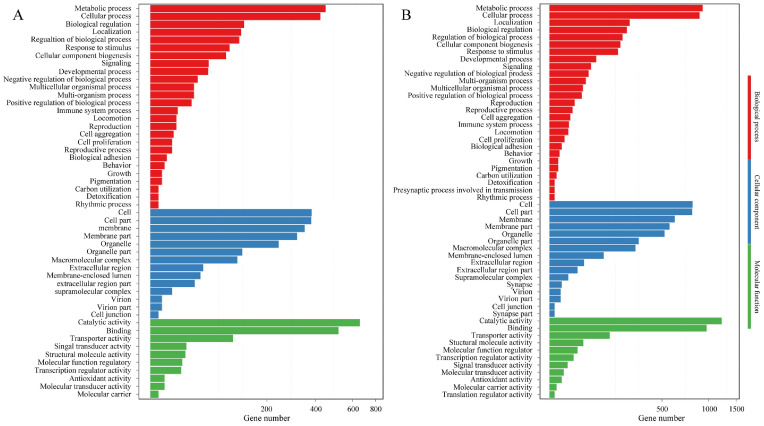
The GO classification of DEGs. (**A**) DEGs between Pd-S and Pd-G; (**B**) DEGs between Pd-S and Pd-M. (**A**) DEGs between Pd-S and Pd-G; (**B**) DEGs between Pd-S and Pd-M. The *x*-axis denotes the number of DEGs, while the *y*-axis lists the specific GO terms. Broader, primary GO categories are color-coded and group their corresponding secondary terms.

**Figure 8:**
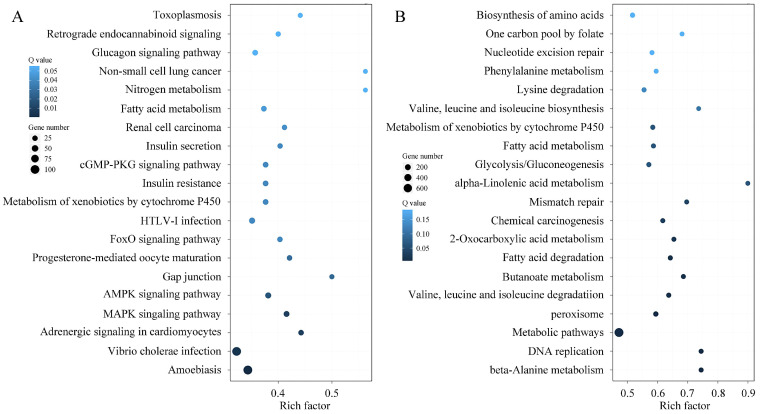
KEGG enrichment analysis of DEGs. (**A**) DEGs between Pd-S and Pd-G; (**B**) DEGs between Pd-S and Pd-M. The *x*-axis denotes the rich factor (the ratio of DEGs to total annotated genes within a given pathway), and the *y*-axis lists the enriched KEGG terms. Data points are presented as bubbles, with their size representing the number of DEGs and their color indicating the corrected *p*-value (Q-value), where a darker color corresponds to a lower Q-value and thus a higher degree of enrichment.

Functional categorization of the DE-mRNAs by GO enrichment highlighted significant involvement in key biological processes, including metabolism, regulation of biological processes, cellular component biosynthesis, development, and reproduction. Analysis of molecular functions further showed that the top five categories, ranked by DE-mRNA count, were catalytic activity, binding, transport, signal transduction, and structural molecule activity (Figure 7). Transcriptional level changes during the spore to hyphae transition reflect a coordinated shift toward activation of virulence, metabolic reprogramming, cell wall remodeling, detoxification, and signaling regulation. KEGG enrichment analysis of DE-mRNAs associated with fungal development highlighted amino acids metabolism, carbohydrate metabolism, fatty acid metabolism, DNA replication, and metabolism of xenobiotics by cytochrome P450, which are closely related to macromolecular biosynthesis and phenotypic changes during fungal development. Several key signaling pathways cGMP-PKG, FoxO, AMPK, and MAPK also play critical roles in regulating fungal growth and secondary metabolism, aligning with established reports [53,54,55,56]. In addition, many DE-mRNAs linked to human diseases were identified during spore germination, likely due to the rapid production of fungal secondary metabolites or mycotoxins, indicating increased virulence in the early stages of germination [57,58]. Moreover, DEGs related to peroxisome indicate the potential involvement of fatty acid β-oxidation, reactive oxygen species scavenging, secondary metabolite production, and conidiation during fungal morphogenesis (Figure 8) [59,60].

**Figure 9:**
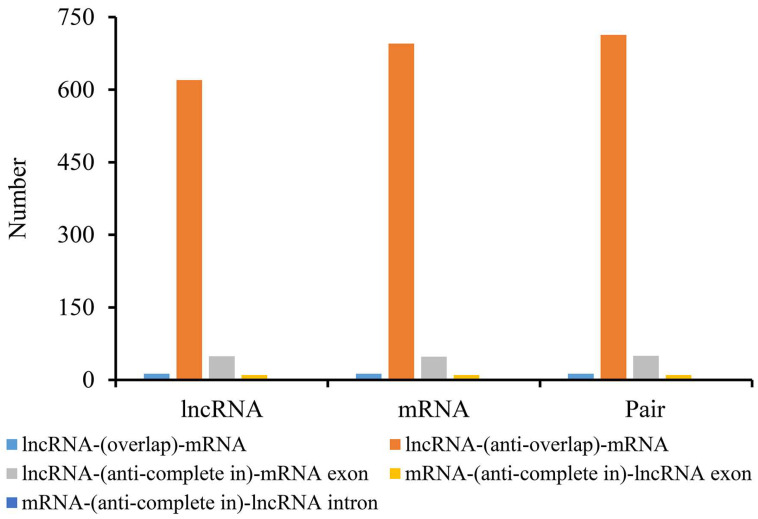
Classification of *cis*-regulatory mRNAs and lncRNAs in *Penicillium digitatum*. The *y*-axis indicates the count of RNAs, while the *x*-axis shows the distinct *cis*-regulatory categories. These categories are color-coded to differentiate among lncRNAs, mRNAs, and lncRNA-mRNA pairs. lncRNA-(overlap)-mRNA: the lncRNA and mRNA in the same chain, and overlap exist (where overlap is a partial overlap, not entirely included in the other one); lncRNA-(anti-overlap)-mRNA: the lncRNA and mRNA in the different chain, and overlap exist (where overlap is a partial overlap, not entirely included in the other one); lncRNA-(anti-complete in)-mRNA exon: the lncRNA and mRNA in the different chain, and the lncRNA is completely in the exon region of mRNA; mRNA-(anti-complete in)-lncRNA exon: the lncRNA and mRNA in the different chain, and the mRNA is completely in the exon region of lncRNA; mRNA-(anti-complete in)-lncRNA intron: the lncRNA and mRNA in the different chain, and the mRNA is completely in the intron region of lncRNA.

**Figure 10:**
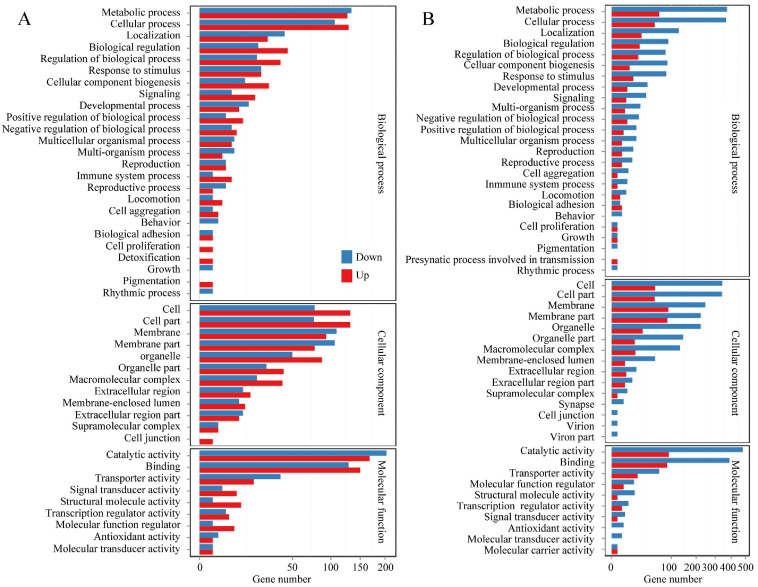
Functional profiling of DE-lncRNA target genes based on GO classification. (**A**) DE-lncRNA target genes between Pd-S and Pd-G; (**B**) DE-lncRNA target genes between Pd-S and Pd-M. The *x*-axis indicates the gene count, while the *y*-axis lists the GO terms. For each term, the stacked bars are color-coded to distinguish between down-regulated and up-regulated target genes.

**Figure 11:**
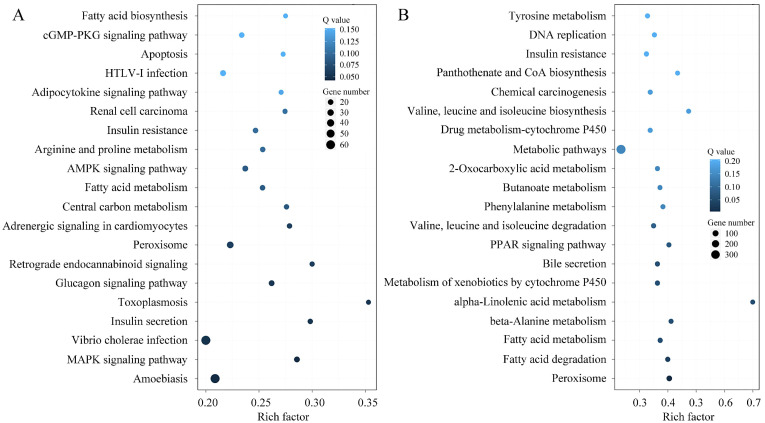
Functional enrichment of DE-lncRNA target genes in KEGG pathways. (**A**) DE-lncRNA target genes between Pd-S and Pd-G; (**B**) DE-lncRNA target genes between Pd-S and Pd-M. The *x*-axis represents the rich factor of DE-lncRNA target genes, and the *y*-axis represents the KEGG terms. The rich factor is the ratio of the number of DE-lncRNA target genes annotated in this KEGG term to the number of total genes annotated in this KEGG term. The bubble size denotes the gene count, while the color gradient corresponds to the corrected *p*-value (Q-value), with darker hues signifying a lower Q-value and a higher degree of enrichment.

The function of lncRNA is related to the protein coding gene near *cis* so as it can serve as a candidate target gene. Therefore, an overlap classification of lncRNAs and their target genes was conducted, and the results were shown in Figure 9 and Table S17. A total of 713 pairs belonged to the type of lncRNA-(overlap)-mRNA, and 50 pairs belonged to the type of lncRNA-(anti-complete in)-mRNA exon. Only one pair was classified as the mRNA-(anti-complete in)-lncRNA intron type. Through *cis* or *trans* way analysis, the target genes of lncRNAs were predicted and the detailed information was provided in Table S18. The GO enrichment results of DE-lncRNA target genes were shown in Figure 10. Notably, in terms of expression trends, the number of upregulated target genes slightly outnumbered downregulated ones between spores and germinated spores, whereas a significantly higher proportion of target genes were downregulated compared to upregulated between spores and mycelia, reflecting dynamic gene expression changes across different developmental stages. The KEGG enrichment profile of DE-lncRNA target genes was high similar to that of the contemporaneous DEGs. Whereas, the profile of DE-lncRNA target genes between spores and germinated spores was significantly different with that between spores and mycelia. The only overlapping items were fatty acid metabolism and peroxisome (Figure 11). This also accommodates the distinct biological significance between spores and mycelia.

### 3.7. Interaction Analysis of lncRNAs-mRNAs and lncRNAs-circRNAs

**Figure 12:**
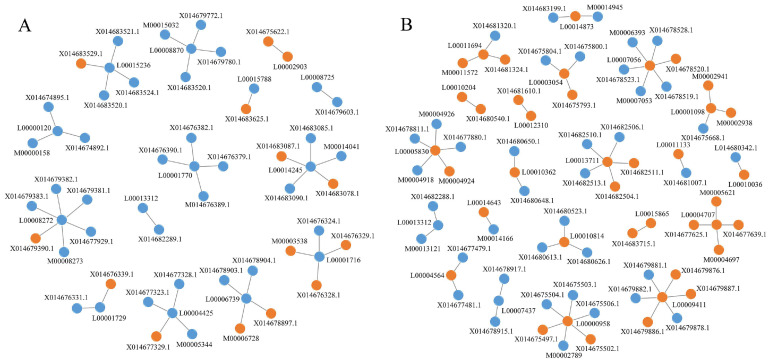
Interaction of DE-lncRNAs and DE-lncRNA target genes in *Penicillium digitatum*. (**A**) interaction analysis between Pd-S and Pd-G; (**B**) interaction analysis between Pd-S and Pd-M. Orange color indicates upregulated and blue color indicates downregulated. The X, M, and L in IDs stand for XM, LTCONS, and MTCONS, respectively.

Through differential expression analysis, the relationship between DE-lncRNAs and DE-lncRNA target genes was extracted. The networks across different developmental stages are displayed in Figure 12. Compared with those during the spores to germination stage, the interaction networks during the spores to mycelia stage were more complicated, and incongruent expression trends between DE-lncRNAs and DE-lncRNA target genes were more frequently observed. It was worth noting that the lncRNA (LTCONS00013312) was present in the interaction networks of both stages. The functions of target genes were related to the peptidoglycan-binding lysin domain and the core regulator of carbon-nitrogen metabolic balance. During spores to germinated spores transition, the molecular network involved multiple functional proteins regulation, including thymine DNA glycosylase (base excision repair), magnesium transporter (ion homeostasis), 12-oxophytodienoate reductase (oxylipin metabolism), myotubularin-related protein (phosphoinositide regulation), NOP2/Sun RNA methyltransferase (RNA modification), serine/threonine-protein phosphatase (signal transduction), NAD(P)H-ubiquinone oxidoreductase (mitochondrial electron transport), retinol dehydrogenase (retinoid metabolism), UTP-glucose-1-phosphate uridylyltransferase (nucleotide sugar biosynthesis), and glucosamine-phosphate N-acetyltransferase (amino sugar metabolism), and others (Figure 12A). The transition from spores to mycelial growth featured distinct network components including spastin (microtubule severing), NADPH:quinone reductase (antioxidant defense), monoacylglycerol lipase (lipid metabolism), DNA-directed RNA polymerase (transcription machinery), glyoxylate reductase (glyoxylate cycle), ubiquitin-like-conjugating enzyme (protein modification), E3 ubiquitin-protein ligase (protein degradation), cytochrome b5 reductase (electron transfer), ATP-dependent RNA helicase (RNA processing), oligosaccharyltransferase (N-glycosylation), and alcohol dehydrogenase (ethanol metabolism), and others (Figure 12B).

**Figure 13:**
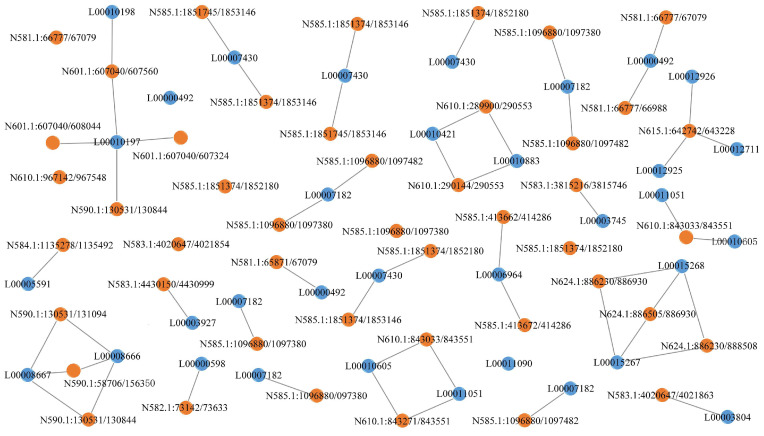
Prediction of the circRNA-lncRNA network in *Penicillium digitatum*. The orange dots represent the circRNAs, and the blue dots represent the lncRNAs. The information on circRNAs and lncRNAs is listed in Table S18. The N in IDs stands for NW014574.

Using Blast to miRBase, we did not find potential miRNA precursors in lncRNAs with a coverage greater than 90%. A total of 144 circRNAs in the lncRNAs were predicted by CIRI, and the detailed information is provided in Table S19. Depending on the location relationship of the circRNAs and lncRNAs, the possible circRNA-lncRNA network was presented in Figure 13. The prediction results indicated that some lncRNAs or circRNAs could participate in multiple interactions.

## 4. Discussion

*P. digitatum* is a significant phytopathogen responsible for postharvest decay in citrus fruits. Understanding the molecular basis of its development and pathogenicity is crucial for devising effective control strategies. RNA transcripts are not merely passive messengers but active drivers of *P. digitatum*’s pathogenic potential. Messenger RNA transcripts direct the synthesis of key virulence factors, including polygalacturonases that macerate the fruit peel and detoxification enzymes that neutralize antifungal compounds. Furthermore, non-coding RNAs provide a layer of sophisticated regulation by fine-tuning the expression of these virulence genes, ensuring a coordinated attack. The responsive transcriptome allows the fungus to adapt to host defenses, such as reactive oxygen species, and efficiently utilize nutrients, ultimately driving the disease cycle from initial colonization to the production of infectious spores. Transcriptomic studies have elucidated specific metabolic adaptations in *P. digitatum*. For instance, under modified atmosphere packaging, the fungus upregulates genes to accelerate glycolysis and the pentose phosphate pathway, promoting oxidative glucose degradation [61]. Additionally, in response to the fungicide prochloraz, numerous transporter genes—including 14 major facilitator superfamily transporters, 8 ATP-binding cassette transporters, and 3 multidrug and toxic compound extrusion family transporters were differentially expressed [62].

Comparative transcriptomics has also yielded considerable insights into the development and pathogenicity of other *Penicillium* species. In *P. expansum*, genes involved in the biosynthesis of ergosterol, organic acids, cell wall-degrading enzymes, and patulin were upregulated in apple tissues and liquid culture [63]. Specific genes, including a concanavalin A-like lectin/glucanase gene (*Peclg*) and LysM family member *PeLysM15*, have been shown to play pivotal roles in fungal growth, virulence, and host–pathogen interactions [64,65]. A comprehensive profiling study in *P. expansum* identified and quantified 3362 lncRNAs, 10 miRNAs, 86 small interfering RNAs (siRNAs), and 11,238 circRNAs across spores and hyphae [66]. In *P. oxalicum*, integrated phenotypic and transcriptome analyses revealed that the transcription factor *PoCrzA* regulates fungal development in a FlbS-BrlA-dependent manner and modulates the expression of cellulolytic genes [67]. Similarly, in *P. citrinum* X9-4, the integration of transcriptome and proteome data demonstrated that ochratoxin A biosynthesis is regulated by ambient pH [68]. During the spoilage of tobacco leaves by *P. citrinum*, genes associated with carbohydrate degradation and the catabolism of fatty acids and aromatic compounds were significantly altered [69]. Furthermore, in *P. italicum*, genes involved in ABC transporters, MFS transporters, ergosterol biosynthesis, mitogen-activated protein kinase signaling, and Ca^2+^/calmodulin-dependent kinase signaling responded to the sterol demethylation inhibitor fungicide prochloraz [70].

Building on previous findings, this study identified 2175 novel mRNA transcripts, along with 12,396 Indels and 23,264 SNPs across different developmental stages from spores to hyphae, utilizing high-throughput RNA sequencing. These genetic variations play pivotal roles in fungal biology. SNPs contribute to genetic variation among fungal populations, aiding in adaptation to different environments, altering virulence factors, resistance against antifungal drugs, and influencing host range [71,72]. Indels drive structural variations, changes in gene function and fungal metabolism, and aid in rapid evolution. The newly discovered transcripts may regulate critical processes such as pathogenicity, fungicide resistance, stress adaptation, and secondary metabolite biosynthesis, presenting potential targets for disease management [73,74]. Additionally, over 8800 AS events were detected per sample. AS is a vital post-transcriptional process in eukaryotes that diversifies the transcriptome and proteome by generating multiple distinct mRNA and protein isoforms from a single gene. In *P. digitatum*, AS likely enhances proteomic complexity, influencing hyphal growth, sporulation, and developmental transitions [75,76].

LncRNAs play crucial regulatory roles in eukaryotic organisms through diverse molecular mechanisms. Depending on cell type, stress conditions, developmental stage, or secondary/tertiary structure, these regulatory molecules can function as molecular signals, miRNA sponges, protein decoys, scaffold RNAs, guide RNAs, enhancer RNAs, antisense RNAs, competing endogenous RNAs, or structural RNAs. Extensive evidence has established lncRNAs as critical regulators in fungal biology [77,78,79]. They can modulate virulence factor expression by interacting with chromatin-modifying complexes or acting as miRNA sponges, suppress host immune responses by interfering with defense signaling pathways, maintain redox homeostasis by regulating peroxisomal genes and ROS-scavenging enzymes, coordinate secondary metabolism as antisense RNAs or molecular scaffolds, control morphological development and sporulation through central developmental pathways, and epigenetically regulate pathogenicity genes via DNA methylation or histone modifications [80,81,82,83]. Additionally, some lncRNAs stabilize virulence-related mRNAs by sequestering their targeting miRNAs, further fine-tuning fungal pathogenicity [84,85]. In *Phytophthora infestans*, lncRNAs may promote asexual development by modulating the expression of key mRNAs involved in development and host invasion, including those encoding INF6, triose-phosphate isomerase, and glycoprotein elicitor. Moreover, lncRNAs could play a role in sexual reproduction by regulating mating-related genes, including M96 mating-specific protein and Crinkler family protein [86]. Likewise, lncRNAs displayed a distinct temporal regulation pattern across developmental stages and showed a significant association with secreted protein and effector genes in *P. sojae* [87]. In *Fusarium graminearum*, the lncRNAs *GzmetE-AS* and *lncRSp1* were identified as functional regulators of asexual and sexual reproduction, achieved through modulating the expression of their respective target genes, *GzmetE* and *Fgsp1* [88,89]. Also, 85 antisense lncRNAs and their respective sense transcripts were induced in parallel as the fruiting bodies matured [90]. Additionally, the lncRNAs *Fo-carP* and *Ff-carp* were positive regulators of carotenoid biosynthesis in *F. oxysporum* and *F. fujikuroi* [91]. In *Pyricularia oryzae*, the expression of lncRNAs was observed to be co-expressed with that of their neighboring genes in both conidia and hyphae. Furthermore, functional analysis implicated one specific lncRNA in hyphal growth, likely through the regulation of an adjacent protein-coding gene. Separately, alternative splicing of the transcription factor gene *CON7* was essential for appressorium formation [92]. During host infection, the lncRNAs regulate pathogenesis-related genes, including xylanases and effectors in *Magnaporthe oryzae* [84]. A coordinated expression program involving 55 antisense lncRNAs and their cognate sense transcripts was activated in *Botrytis cinerea* during the invasion of tomato [79]. In *Blumeria hordei*, lncRNAs also played a compensatory role by enhancing transcriptional diversity and plasticity, thereby serving as key drivers of rapid evolutionary adaptation that bolstered pathogenicity and facilitated the overcoming of host defenses [93]. Furthermore, the lncRNAs in *Aspergillus flavus* were proposed to function as multifunctional hubs, centrally regulating aflatoxin production, respiratory metabolism, cell survival, and stress adaptation [78]. While research on lncRNAs in *P. digitatum* remains limited compared to model fungi.

This study predicted a total of 3957 novel lncRNAs, categorizing them into known conserved families based on evolutionary features. These lncRNAs are distributed across various genomic regions with distinct positional patterns, including intergenic lncRNAs, antisense lncRNAs, intronic lincRNAs, and promoter-associated lncRNAs. Through transcriptional regulation, post-transcriptional control, and stability regulation, lncRNAs and mRNAs engage in complex regulatory interactions that fine-tune gene expression. To elucidate their functional roles, target genes of lncRNAs were predicted based on *cis-* or *trans*-acting modes, and their expression levels were determined. GO and KEGG enrichment analyses of DE-target genes across different developmental stages of *P. digitatum* revealed patterns similar to those of DEGs. Furthermore, differential expression analysis explored interactions between DE-lncRNAs and their target genes, with a regulatory network visualized in Figure 12. Additionally, the miRNA precursors and circRNAs we screened in the lncRNAs. A total of 144 circRNAs were successfully identified, while no miRNA precursors met the established screening thresholds. CircRNAs represent a distinct category of endogenous non-coding RNAs, distinguished by their covalently closed continuous loop structure, which results from a non-canonical back-splicing event in pre-mRNA [27,28]. Unlike linear RNAs, circRNAs lack 5′ caps and 3′ poly(A) tails, conferring high stability and resistance to exonucleases. LncRNAs and circRNAs represent two important classes of non-coding RNAs with distinct yet complementary roles in gene regulation. LncRNAs often modulate splicing factors and regulate cicrRNA biogenesis. The circRNAs derived from lncRNA loci could co-regulate the common target pathway. Both of them can act as miRNA sponges and compete for shared miRNA binding sites [94,95]. Their potential interactions within cellular networks were summarized in Figure 13, which is an important layer of gene regulation, influencing the development and pathogenicity of *P. digitatum.*

The pathogenicity of *P*. *digitatum*, the causal agent of citrus green mold, is closely linked to its developmental stages (spore germination, hyphal growth, and sporulation), which determine its ability to infect, colonize, and spread. Transcriptomic analysis of stage-specific lncRNAs and mRNAs reveals key regulatory networks controlling growth and virulence factors in the current work. Future studies should prioritize the functional characterization of these novel transcripts to further elucidate their roles in *P. digitatum* biology. These molecular insights would empower innovative approaches for combating *P. digitatum* infections through targeted interventions. For example, citrus cultivars engineered with host-induced gene silencing constructs demonstrate enhanced resistance by disrupting fungal gene networks. CRISPR-based genome editing tools are deployed to disrupt master developmental regulators that coordinate pathogenic progression. Nano-formulated double-stranded RNAs offer precise silencing of virulence-associated lncRNAs and mRNAs critical for fungal germination and hyphal propagation. Complementary to these genetic strategies, structure-guided small molecule inhibitors provide chemical interference with lncRNA-mediated pathogenic pathways and quorum-sensing mechanisms. The integration of these approaches will facilitate the creation of stage-specific, environmentally friendly solutions that effectively mitigate postharvest disease losses in citrus production.

## 5. Conclusions

This study uncovered profound molecular dynamics of *P. digitatum* underlying its pathogenic lifecycle. The developmental transitions from spores to germinated spores and finally to mycelia are orchestrated by extensive transcriptomic reprogramming, evidenced by the identification of thousands of novel mRNAs and lncRNAs, stage-specific differential expression of over 2000–4000 genes, and widespread AS events. These signatures highlight a coordinated shift in fungal physiology, activating major regulatory pathways that are central to pathogenicity, including MAPK and cAMP signaling for developmental coordination, macromolecular metabolic pathways to fuel growth and infection structure formation, and robust detoxification mechanisms, such as reactive oxygen species scavenging, to overcome host defenses. Crucially, the construction of lncRNA-mRNA interaction networks revealed an additional layer of sophisticated gene regulation, fine-tuning these processes. These findings provide a holistic view of the pathogen’s virulence network, unveiling a rich repository of stage-specific molecular targets. This opens the door for pioneering, RNA-level citrus disease management strategies, such as host-induced gene silencing or nano-formulated RNAi sprays, designed to disrupt these critical pathways and suppress fungal growth and virulence with high precision and sustainability.

## Data Availability

The data used to support the findings of this study can be made available by the corresponding authors upon request.

## Supplementary Materials

The following supporting information can be downloaded at https://www.mdpi.com/article/10.3390/microorganisms13122879/s1: Figure S1 Amplified products using the ITS1/ITS4, ITS4/ITS5, and BT2a/BT2b primer pairs; Figure S2: Circular phylogenetic tree constructed from the top 20 BLAST hits; Figure S3: Growth dynamic detection of *Penicillium digitatum*; Figure S4: The annotation of mRNA genes in spores, germinated spores and mycelia of *Penicillium digitatum*; Figure S5: LncRNA family analysis; Figure S6: The GO enrichment analysis of differential splicing genes (DSGs); Figure S7: The density map of the mRNAs and lncRNAs expression levels in spores (A), germinated spores (B) or mycelia (C) of *Penicillium digitatum*; Figure S8: Clustering results of temporal expression pattern of the lncRNAs and mRNAs during different developmental stages of *Penicillium digitatum*; Figure S9: The clustering of DE-lncRNAs and DE-mRNAs of *Penicillium digitatum* at different developmental stages; Table S1: Amplified products using the ITS1/ITS4, ITS4/ITS5, and BT2a/BT2b primer pairs; Table S2: The information of the primer pairs used for qRT-PCR; Table S3: Filtering and assembling of the data; Table S4: The coding capacity prediction of *Penicillium digitatum* transcripts across different developmental stages; Table S5: The sequence information of the novel mRNAs in *Penicillium digitatum*; Table S6: The sequence information of the novel lncRNAs in *Penicillium digitatum*; Table S7: Annotation of all mRNAs by Non-Redundant Protein Database (NR) of NCBI; Table S8: Annotation of all mRNAs by Gene Ontology (GO) database; Table S9: Annotation of all mRNAs by Kyoto Encyclopedia of Genes and Genomes (KEGG) database; Table S10: Annotation of all mRNAs by Clusters of Orthologous Groups (COGs) database of NCBI; Table S11: Family annotation of the lncRNAs in *Penicillium digitatum*; Table S12: Single nucleotide polymorphism (SNP) analysis of *Penicillium digitatum* across developmental stages; Table S13: Insertion-Deletion (InDel) analysis of *Penicillium digitatum* across different developmental stages; Table S14: Alternative splicing (AS) analysis of *Penicillium digitatum* across different developmental stages; Table S15: The expression levels of transcripts in *Penicillium digitatum* at different developmental stages; Table S16: The differentially expressed genes between different developmental stages in *Penicillium digitatum*; Table S17: Overlap classification existing on the lncRNAs and the target genes in *Penicillium digitatum*; Table S18: LncRNA target genes prediction in *Penicillium digitatum*; Table S19: CircRNAs prediction in *Penicillium digitatum* lncRNAs of at different developmental stages.

## Abbreviations

The following abbreviations are used in this manuscript:

*P. digitatum*	*Penicillium digitatum*
Pd-S	Spores of *P. digitatum*
Pd-G	Germinated spores of *P. digitatum*
Pd-M	Mycelia of *P. digitatum*
lncRNA	Long non-coding RNA
qRT-PCR	Quantitative real-time PCR
DEGs	Differentially expressed genes
SNPs	Single Nucleotide Polymorphisms
INDEL	Insertion-Deletion
GO	Gene Ontology
KEGG	Kyoto Encyclopedia of Genes and Genomes
