# Supplementary material for: Transcriptomic Profiling of mRNA and lncRNA During the Developmental Transition from Spores to Mycelia in Penicillium digitatum
Source: Microorganisms. 2025 Dec 18;13(12):2879. doi: 10.3390/microorganisms13122879 (PMC12736287; doi:10.3390/microorganisms13122879)
Supplement: Supplementary file 1 [file microorganisms-13-02879-s001.zip › Supplementary materials.pdf]

Transcriptomic Profiling of mRNA and lncRNA during the Developmental Transition from Spores to Mycelia in *Penicillium digitatum*

Ting Zhou [1], Yajie Yang [1], Fei Wang [1], Linqian Liang [1], Ziqi Zhang [1],Heru Dong [1], Zhaocheng Jiang [1], Pengcheng Zhang [1],* and Tongfei Lai [1,]*

[1] College of Life and Environmental Science, Hangzhou Normal University, Hangzhou 310036, China; 20100061@hznu.edu.cn (T.Z.); 2024111010034@stu.hznu.edu.cn (Y.Y.); 2025111010051@stu.hznu.edu.cn (F.W.), 2025112010055@stu.hznu.edu.cn (L.L.); 2023210301069@stu.hznu.edu.cn (Z.Z.); 2023210301042@stu.hznu.edu.cn (H.D); 2023210301051@stu.hznu.edu.cn (Z.J.)

*Correspondence: laitongfei@hznu.edu.cn (T.L.); zpc604@hznu.edu.cn (P.Z.)

Supplementary Materials:

Supplementary tables

Table S1 Amplified products using the ITS1/ITS4, ITS4/ITS5, and BT2a/BT2b primer pairs

Table S2 The information of the primer pairs used for qRT-PCR

Table S3 Filtering and assembling of the data

Table S4 The coding capacity prediction of *Penicillium digitatum* transcripts across different developmental stages

Table S5 The sequence information of the novel mRNAs in *Penicillium digitatum*

Table S6 The sequence information of the novel lncRNAs in *Penicillium digitatum*

Table S7 Annotation of all mRNAs by Non-Redundant Protein Database (NR) of NCBI

Table S8 Annotation of all mRNAs by Gene Ontology (GO) database

Table S9 Annotation of all mRNAs by Kyoto Encyclopedia of Genes and Genomes (KEGG) database

Table S10 Annotation of all mRNAs by Clusters of Orthologous Groups (COGs) database of NCBI

Table S11 Family annotation of the lncRNAs in *Penicillium digitatum*

Table S12 Single nucleotide polymorphism (SNP) analysis of *Penicillium digitatum* across developmental stages

Table S13 Insertion-Deletion (InDel) analysis of *Penicillium digitatum* across different developmental stages

Table S14 Alternative splicing (AS) analysis of *Penicillium digitatum* across different developmental stages

Table S15 The expression levels of transcripts in *Penicillium digitatum* at different developmental stages

Table S16 The differentially expressed genes between different developmental stages in *Penicillium digitatum*

Table S17 Overlap classification existing on the lncRNAs and the target genes in *Penicillium digitatum*

Table S18 LncRNA target genes prediction in *Penicillium digitatum*

Table S19 CircRNAs prediction in *Penicillium digitatum* lncRNAs of at different developmental stages

Supplementary figures:

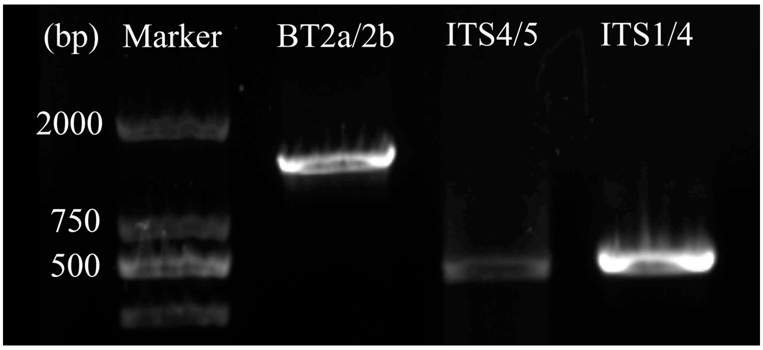

Figure S1 Amplified products using the ITS1/ITS4, ITS4/ITS5, and BT2a/BT2b primer pairs.

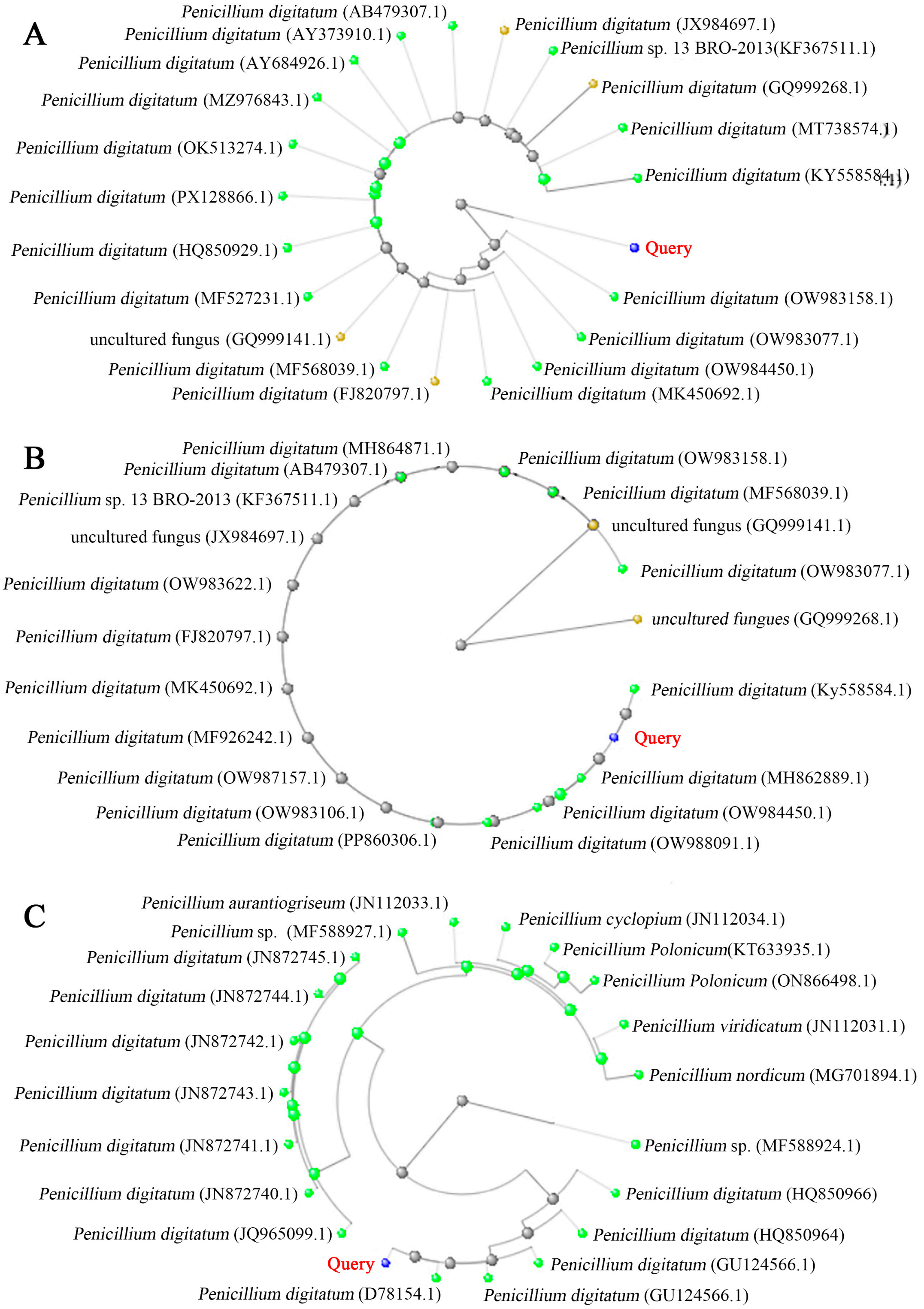

Figure S2 Circular phylogenetic tree constructed from the top 20 BLAST hits. The tree is built using the Fast Minimum Evolution method based on BLAST pairwise alignments. The query sequences are generated by PCR amplification using the primer

pairs ITS1/ITS4 (A), ITS4/ITS5 (B), and BT2a/BT2b (C). The blue node represents the isolate used in this study, the green node indicates fungi, the yellow node denotes ascomycete fungi, and the grey node corresponds to taxa identified as both fungi and ascomycetes. The taxonomic name (sequence ID) is labeled beside the corresponding node.

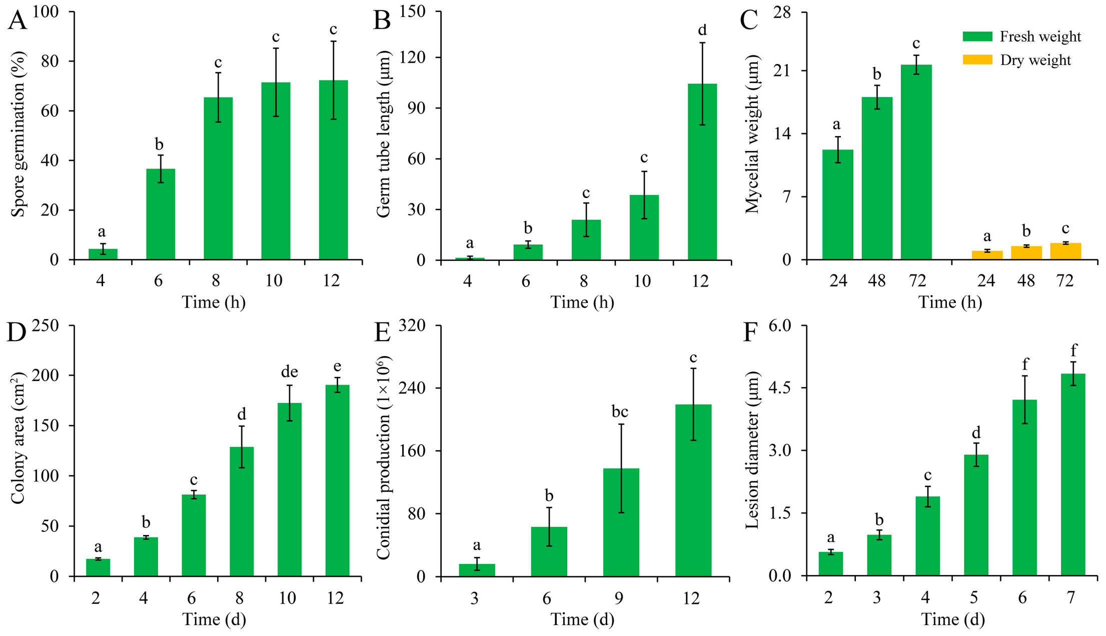

Figure S3 Growth dynamic detection of *Penicillium digitatum.* (A) spore germination; (B) germ tube length; (C) mycelial accumulation; (D) colonial expansion; (E) sporulation; (F) lesion formation induced by *P. digitatum* on circus fruit.

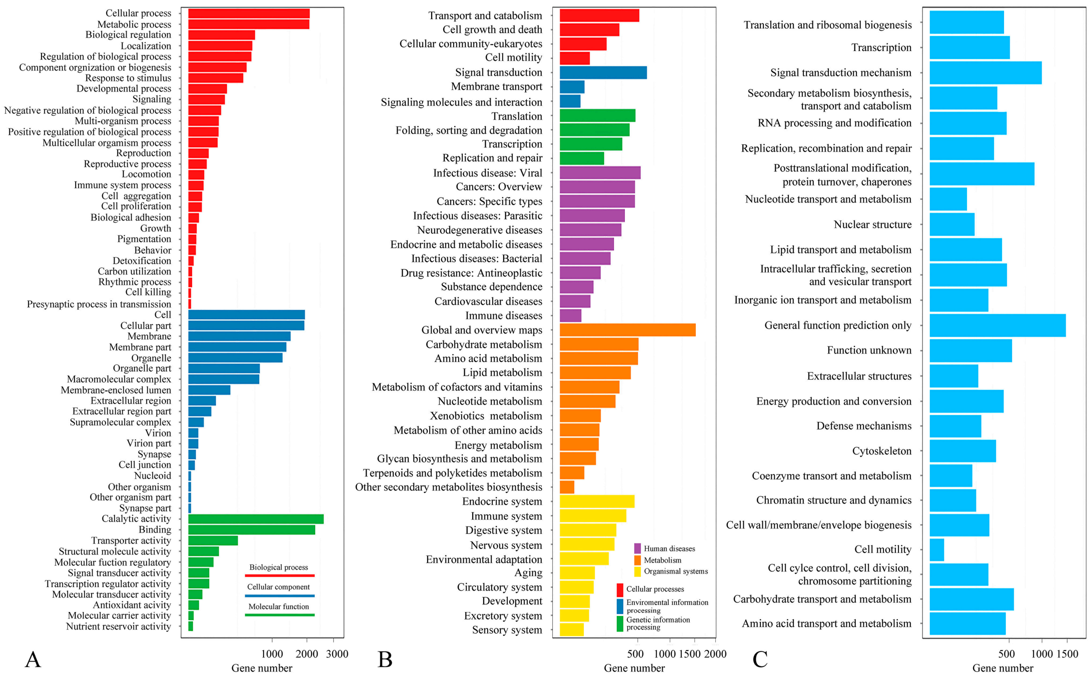

Figure S4 The annotation of mRNA genes in spores, germinated spores and mycelia of *Penicillium digitatum*. (A) GO annotation statistics; (B) KEGG annotation statistics; (C) COG annotation statistics. The x axis represents the number of genes, and the y axis represents the GO, KEGG or COG entry. The different color indicates the different classification of GO or KEGG.

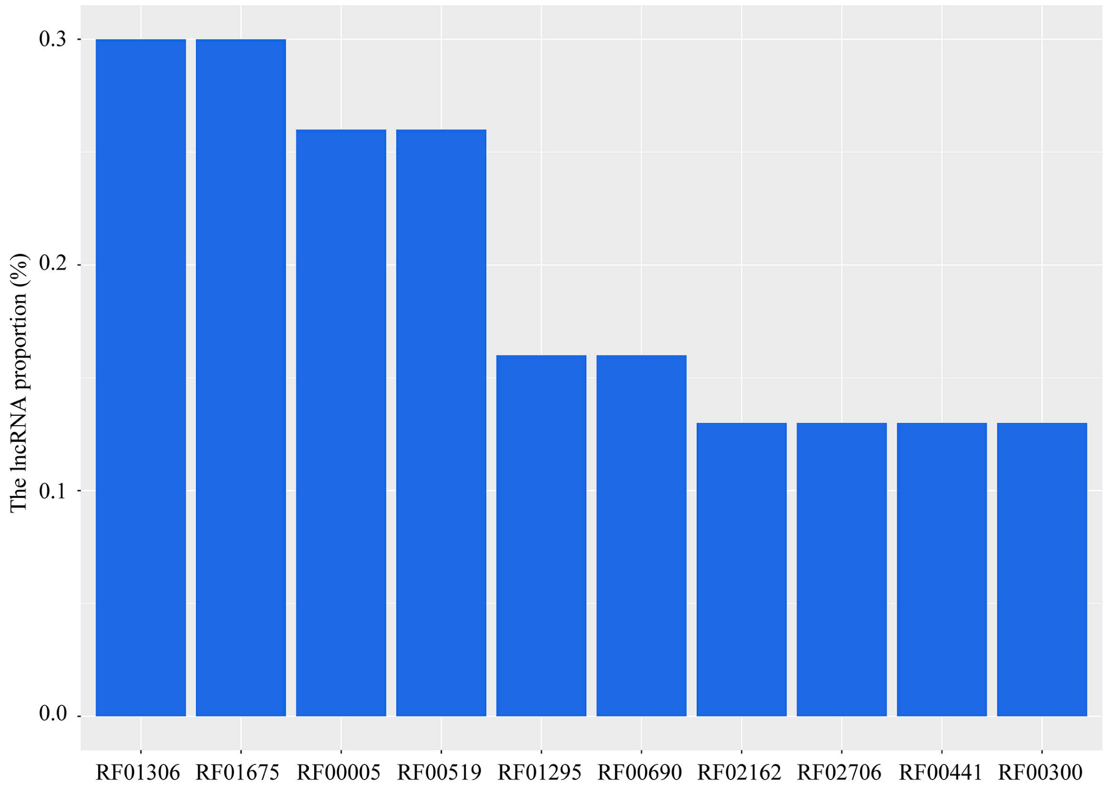

Figure S5 LncRNA family analysis. The x axis indicates the lncRNA family and the y axis indicates the lncRNA number.

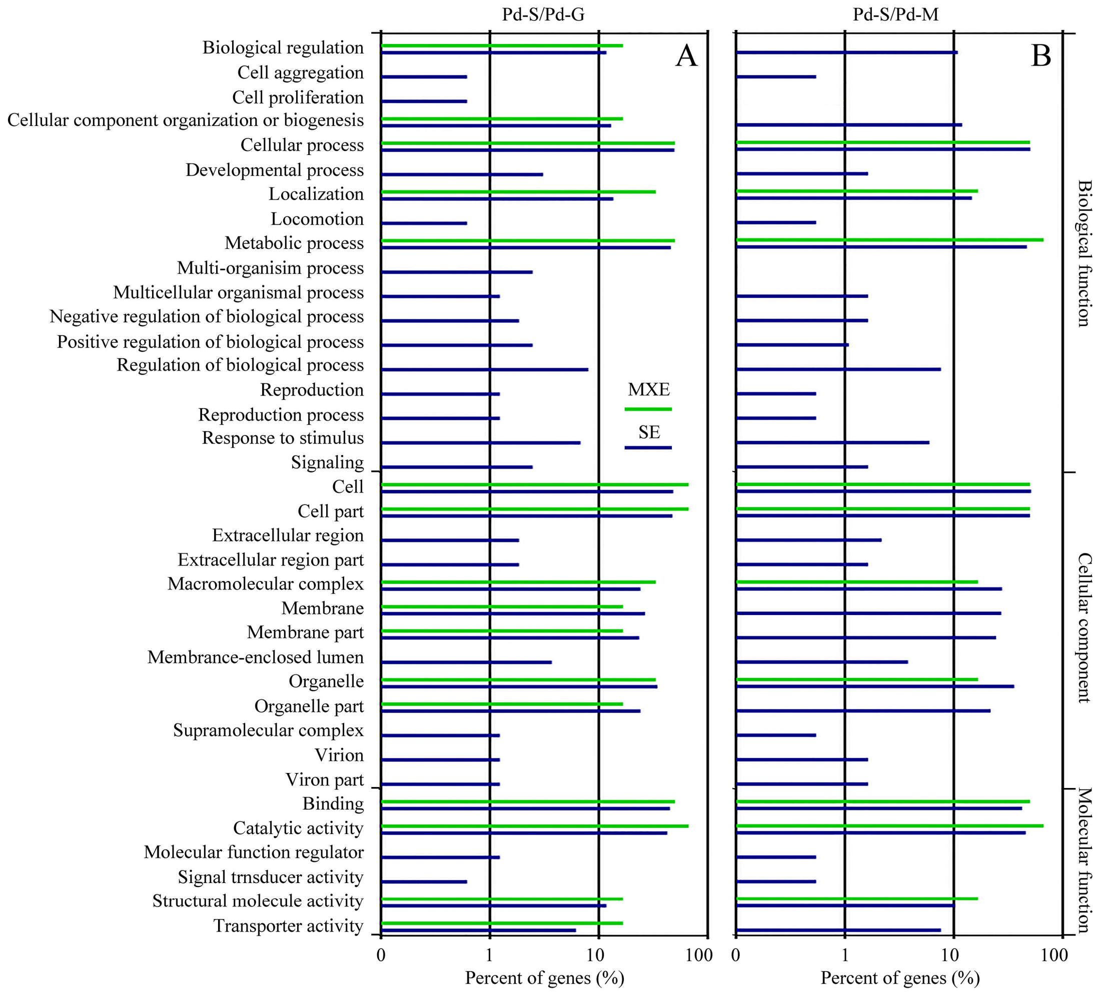

Figure S6 The GO enrichment analysis of differential splicing genes (DSGs). (A) DSGs between Pd-S and Pd-G; (B) DSGs between Pd-S and Pd-M. The x axis represents the ratio of the DSGs involved in a particular GO category to all DSGs. The y axis represents the GO category. MXE: mutually exclusive exons; SE: skipped exon.

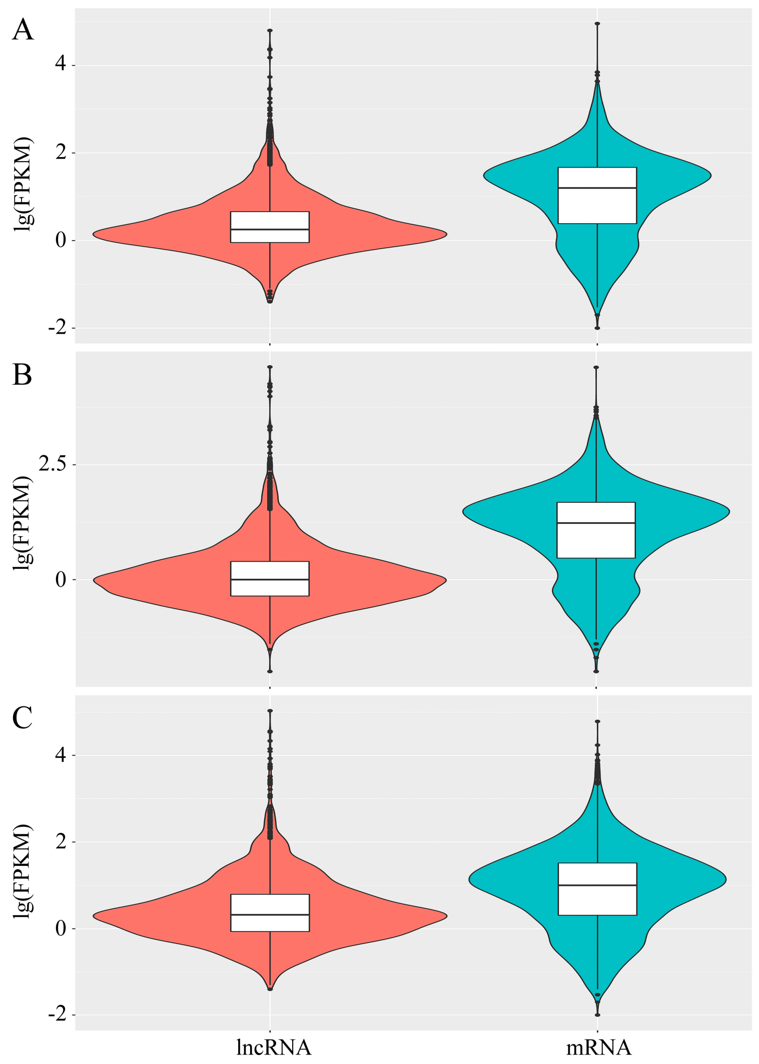

Figure S7 The density map of the mRNAs and lncRNAs expression levels in spores (A), germinated spores (B) or mycelia (C) of *Penicillium digitatum*. The x axis indicates the RNA density of a certain expression, the y axis indicates the value of lg(FPKM), and in the middle there is box plot. From bottom of the figure there is: the lower quarter, the median (black in thick line) and the upper quarter. The median is the number in the middle positions in the data, that is, half of the data is greater than the median (above), the other half is less than the median (below). A quarter of the data is greater than the upper quartile, which is above the white box; another quarter of the data is less than the lower quartile, which is under the white box.

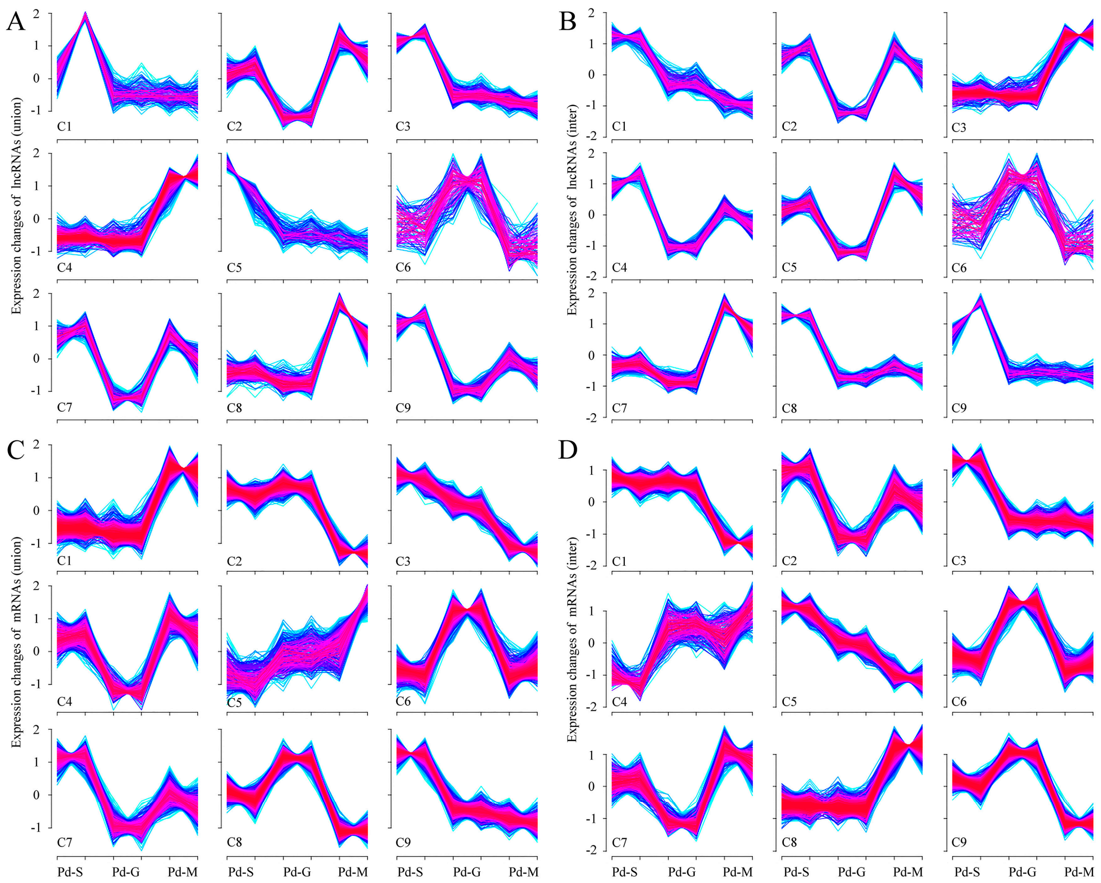

Figure S8 Clustering results of temporal expression pattern of the lncRNAs and mRNAs during different developmental stages of *Penicillium digitatum*. (A) the lncRNAs in the union set; (B) the lncRNAs in the inter set; (C) the mRNAs in the union set; (D) the mRNAs in the inter set. The inter set requires that the gene express in all samples, and the union set requires only express in one sample. C1 to C9 indicate nine patterns of clustering results. Membership values are color-encoded with red and purple shades denoting high membership values, and dark blue and light blue shades denoting low membership values of genes.

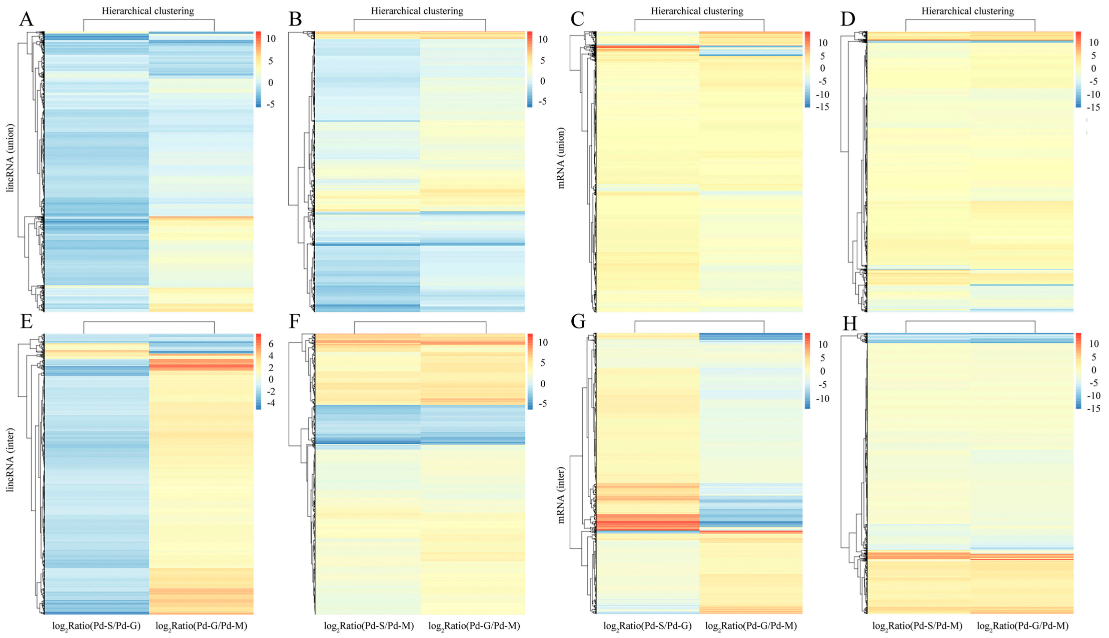

Figure S9 The clustering of DE-lncRNAs and DE-mRNAs of *Penicillium digitatum* at different developmental stages. (A) union of DE-lncRNAs between Pd-S/Pd-G and Pd-G/Pd-M; (B) union of DE-lncRNAs between Pd-S/Pd-M and Pd-G/Pd-M; (C) union of DE-mRNAs between Pd-S/Pd-G and Pd-G/Pd-M; (D) union of DE-mRNAs between Pd-S/Pd-M and Pd-G/Pd-M; (E) intersection of DE-lncRNAs between Pd-S/Pd-G and Pd-G/Pd-M; (F) intersection DE-lncRNAs between Pd-S/Pd-M and Pd-G/Pd-M; (G) intersection of DE-mRNAs between Pd-S/Pd-G and Pd-G/Pd-M; (H) intersection of DE-mRNAs between Pd-S/Pd-M and Pd-G/Pd-M. The x axis indicates clustering scheme, the y axis indicates the RNAs, and the color represents clustering value. The inter set requires that the genes express in all samples, and the union set requires that the genes only express in one sample.
